# Magnetic field sensing subject to correlated noise with a ring spin chain

**DOI:** 10.1038/srep33254

**Published:** 2016-09-13

**Authors:** Li-Sha Guo, Bao-Ming Xu, Jian Zou, Bin Shao

**Affiliations:** 1 School of Physics, Beijing Institute of Technology, Beijing 100081, China; 2 School of Physics, Qufu Normal University, Qufu 273165, China

## Abstract

In this paper, we focus on the magnetic field sensing subject to a correlated noise. We use a ring spin chain with only the nearest neighbor interactions as our probe to estimate both the intensity *B* and the direction *θ* of the magnetic field when the probe reaches its steady state. We numerically calculate the quantum Fisher information (QFI) to characterize the estimation precision. On the one hand, for estimating *B*, we find that the coupling between spins in the probe plays an important role in the precision, and the largest value of the QFI can be achieved when *θ* = *π*/2 together with an optimal coupling. Moreover, for any direction, the precision scaling can be better than the Heisenberg-limit (HL) with a proper coupling. On the other hand, for estimating *θ*, we find that our probe can perform a high precision detection for *θ* ~ *π*/2, with the QFI much larger than that for any other directions, especially when the coupling is tuned to the optimal value. And we find that the precision scaling for *θ* ~ *π*/2 can be better than the HL, but for other directions, the precision scaling is only limited to the standard quantum limit (SQL). Due to the computational complexity we restrict the number of spins in the probe to 60.

Quantum metrology is a fundamental and important subject in physics, which uses entanglement to increase the precision of parameter estimation by quantum measurements beyond the limit of its classical counterpart, with its widely applications in quantum frequency standards^1^,^2^, optical phase estimation^3^,^4^,^5^,^6^,^7^,^8^,^9^, atomic clocks^10^,^11^,^12^,^13^,^14^, atomic interferometers^15^, and magnetic field sensing^16^,^17^. The ultimate precision limit restricted by the quantum mechanics is lower bounded by the quantum Cramér-Rao bound^18^, which is proportional to the inverse of the square root of the so-called quantum Fisher information (QFI). In an ideal setting without noise, it is well known that when the particles are classically correlated and noninteracting, the best scaling achievable can reach the standard quantum limit (SQL) as a consequence of the central limit theorem, i.e., QFI ∝ *N*, where *N* is the number of particles in a probe. However, with the assistance of *N*-particle entanglement, the best precision scaling can arrive at the Heisenberg-limit (HL), i.e., QFI ∝ *N*^2^, implying a quadratic improvement in the scaling of QFI over the SQL^19^,^20^,^21^,^22^. Moreover, when the Hamiltonian is nonlinear or there are interactions among *N* particles, the best scaling can even surpass the HL^23^,^24^. All of the above studies are about the dynamical estimation, i.e., the probe state used for estimation changes with time. However, there is another on-going interest to perform a high precision estimation using the ground (thermal) state, which is associated with the quantum phase transitions^25^,^26^,^27^,^28^,^29^,^30^.

In reality, any real quantum system is inevitably affected by its surrounding environment which impairs the metrological capabilities, reducing the quadratic improvement drastically. It has been shown in the seminal work on noisy quantum metrology that for the uncorrelated Markovian dephasing noise the product and maximally entangled states become asymptotically equivalent, and even the optimal partially entangled states with a highly symmetry can only improve the quantum enhancement to a constant factor, and thus bound the precision scaling to the standard quantum limit SQL^1^. Henceforth, a lot of efforts have been devoted to the uncorrelated noisy metrology^16^,^17^,^31^,^32^,^33^,^34^,^35^,^36^,^37^. And a super-classical precision scaling *N*^−3/2^ or *N*^−5/3^ has been found for the non-Markovian dephasing noise^16^,^17^,^36^ or a preferred transversal direction noise^35^, respectively. Nevertheless, studies on correlated noisy metrology have been relatively few up to now and most of them focused on the correlated dephasing noise^38^,^39^,^40^. It has been shown that only marginal quantum improvements can be achieved in the presence of correlated dephasing noise and the best precision scaling has been reduced below the SQL. However, when the temporal correlation has been considered, the best precision scaling using a highly entangled state can reach the SQL^38^. Besides, by making use of decoherence free subspaces^39^ or higher-dimensional probe systems^40^, quantum enhancement can readily be achieved and can even restore the HL. Apart from the above dynamical estimation in the presence of noise, steady state has aroused some attentions to be as a resource for parameter estimation quite recently^41^,^42^,^43^. Specifically, ref. 41 has studied the QFI scaling by using the nonequilibrium steady state of an XXZ spin chain with local dissipation at its ends as a probe for the parameter estimation, and a superlinear scaling has been found close to the isotropic limit (XXX interaction) and in the easy-plane anisotropic phase, and a superexponential scaling has been found in the easy-axis anisotropic phase. Besides, ref. 42 has shown that by using a stationary state created by correlated dephasing noise, an improvement in the precision not in the scaling compared to standard Ramsey interferometry can be gained. Moreover, ref. 43 has investigated the QFI of a steady state of two qubits in the presence of a local dephasing noise with a reset machanism, and has shown that by choosing a proper coupling strength, the QFI scaling can be larger than the SQL at a certain value of the reset and decoherence parameters.

And in this paper, we use a steady state in the presence of a correlated dissipative Markovian noise to estimate an unknown magnetic field. Specifically, we use a ring spin chain consisting of *N* spins, with only the nearest neighbor interactions, as our probe to estimate the intensity *B* and the direction *θ* of an external magnetic field when the probe reaches its steady state. We first study the different factors influencing the value of QFI, then investigate the precision scaling for estimating *B* and *θ*, respectively.

Our main results are as follows: On the one hand, for estimating *B*, both the coupling between spins in the probe Ω and the direction of magnetic field *θ* have an effect on the value of QFI, and the largest value of QFI can be achieved when *θ* = *π*/2 together with an optimal coupling. In addition, we find that the precision scaling is dependent on Ω, and for any direction *θ*, the precision scaling with a proper coupling can be better than the HL. On the other hand, for estimating *θ*, the coupling also plays an important role in the precision, moreover, the value of QFI is strongly dependent on *θ*. Specifically, our probe can perform a high precision detection for *θ* ~ *π*/2, with the QFI much larger than that for any other directions, especially when the coupling is tuned to the optimal value. In addition, we find that the precision scaling is also dependent on *θ*. When *θ* ~ *π*/2, the scaling can be better than the HL, but when *θ* is not equal or not close to *π*/2, the scaling is just constrained near the SQL, no matter what the value of coupling is.

## Results

### Model

**Figure 1:**
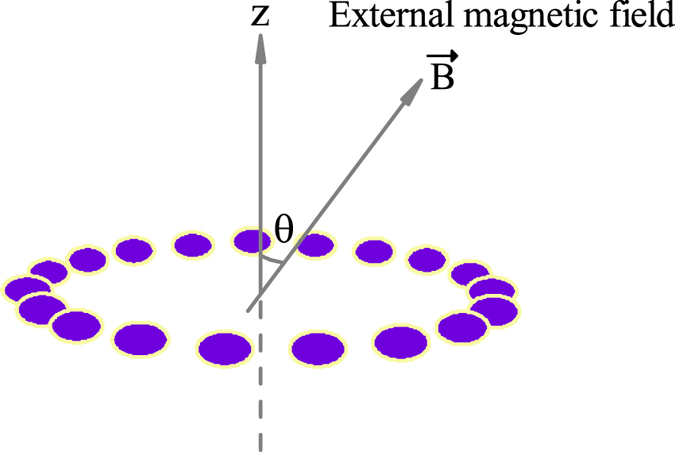
A schematic diagram showing the magnetic field sensing with a ring spin chain with only the nearest neighbor interactions. 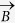
 represents the unknown external magnetic field, and the *z* axis is perpendicular to the plane defined by the ring spin chain. *θ* represents the angle between the *z* axis and the magnetic field.

In this paper, we use a ring spin chain consisting of *N* spins, with only the nearest neighbor interactions, as our probe to estimate an unknown magnetic field in the presence of correlated dissipative Markovian noise (see Fig. 1). It is noted that besides the collective dephasing noise (a dominant source of noise in experiments based on trapped ions), this collective dissipative noise does exist in some relevant experiments. For example, the superradiance phenomenon can occur when an ensemble consisting of *N* identical sub-systems collectively interact with a common radiation field^44^,^45^, whose dynamics can be derived as a collective dissipative Markovian noise. Up to now the superradiance phenomenon has been observed in the semiconductor quantum dot^46^, crystal defects and molecules^47^, and cold atomic gases^48^. The Hamiltonian of the probe exposed to the external magnetic field can be written as


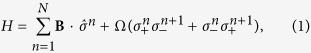


where **B** = *B*(sin *θ* cos *ϕ*, sin *θ* sin *ϕ*, cos *θ*) and 
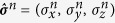
, with *B* representing the intensity of magnetic field, and *θ* and *ϕ* representing the direction of magnetic field, and without losing generality we assume *ϕ* = 0. Here *B* and *θ* are the two parameters we want to estimate in this paper. Besides, 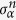 (*α* = *x, y, z*) and 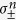 are the usual Pauli operators defined with respect to the *n*th spin of the probe, with the periodic boundary condition 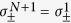. *N* is the number of spins in the probe, and Ω is the coupling between any adjacent two spins. It is noted that the similar ring structure probe can also be used to estimate the temperature of a bath^49^.

For the convenience of discussion in the following we rewrite the probe Hamiltonian *H* as


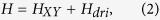


where we name 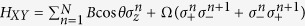 the *XY* spin chain Hamiltonian under the influence of magnetic field and 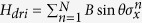 the driving Hamiltonian. By defining the total angular momentum operators 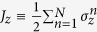 and 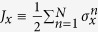, we can rewrite *H*_*XY*_ as


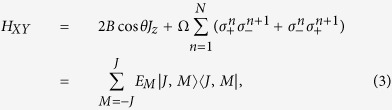


where


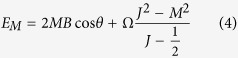


is the eigenenergy of *H*_*XY*_ corresponding to the eigenvector |*J, M*〉 (the Dicke state) with *J* = *N*/2 being fixed, and 2*MB* cos *θ* (the first term in Eq. (4)) represents the modified energy caused by the external magnetic field. And the detailed derivation of Eq. (4) has been shown in refs 44,45. And in order to make the analyses in the next section easy to understand, we call *E*_*M*_ as the ‘energy levels’, but they are not truly the energy levels of probe Hamiltonian *H* (Eq. (1)). Besides, the driving Hamiltonian can be rewritten as


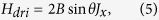


which drives the probe up the |*J, M*〉 ladder and the driving strength 2*B* sin *θ* increases with *θ*.

The dynamical evolution of the probe under the correlated dissipative Markovian noise can be described by the following master equation


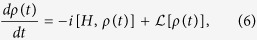


with the Lindblad operator^44^


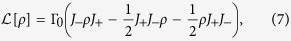


where 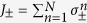, and 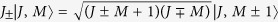. And Γ_0_ is the decoherence rate, which cascades the probe down the |*J, M*〉 ladder to the ground state |*J*, −*J*〉 (*M* = −*J*). From this point, if there is no external magnetic field, the probe would eventually evolve to the ground state |*J*, −*J*〉. And if the external magnetic field is applied and the direction *θ* = 0, i.e., *H*_*dri*_ = 0 (Eq. (5)), only the energy levels *E*_*M*_ would make a modification (i.e., 2*MB*) but the eigenvectors |*J, M*〉 would not change, so the probe would also eventually evolve to the ground state |*J*, −*J*〉, without any information about the unknown magnetic field. However, if the external magnetic field is applied and the direction *θ* ≠ 0, the driving Hamiltonian *H*_*dri*_ ≠ 0, and in this case the driving caused by the magnetic field would compete with the decoherence to some extent and finally make the probe get populated not only in the ground level, hence the probe can obtain a certain amount of information about the unknown magnetic filed.

Besides, since we are interested in the magnetic field sensing when the probe evolves to the steady state, we should just solve the equation


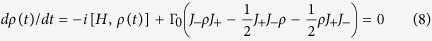


to obtain the stationary solutions. According to our calculation, we obtain the time that the probe arrives at the steady state 
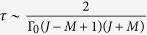
. And the maximum of *τ* (when *M* = *J*) is proportional to 1/*N*, i.e., 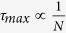. Due to the fact that it is very hard to solve Eq. (8) analytically, we solve it numerically in the total angular momentum representation |*J, M*〉 (*M* = −*J*, …, *J*), and our numerical method allow us to investigate the system up to 60 spins. Here it should be noted that the steady state is only dependent on the arguments in the probe Hamiltonian *H* and is independent of what the initial state we choose, so that a sophisticated preparation of special initial state is not needed. And based on the stationary solutions, we can further calculate the QFI of the probe using the parameter estimation theory (see Methods).

### Estimation of intensity and direction of magnetic field

In this section, we first discuss the estimation of *B* and then the estimation of *θ*, by studying the different factors influencing the value of QFI and the precision scaling, respectively.

### Estimating intensity *B*

We begin with studying the effect of *θ* and Ω on the QFI of estimating *B*, i.e., QFI_*B*_. According to Eq. (12) (see Methods), from our numerical calculations, we find that the largest value of QFI_*B*_ can be achieved when 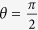 together with an optimal coupling.

**Figure 2:**
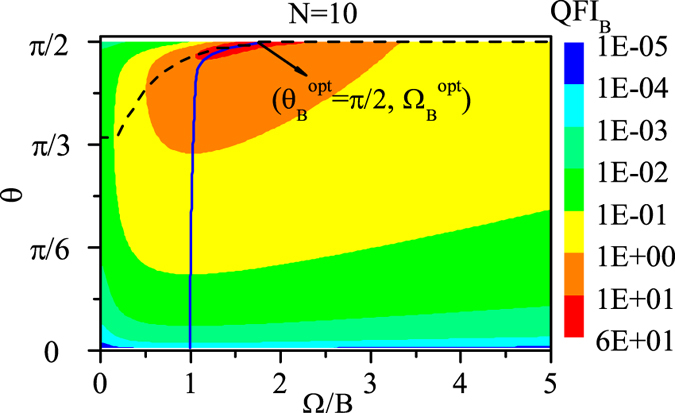
QFI_*B*_ as functions of the direction *θ* and coupling Ω with *N* = 10. The black dashed line represents the optimal *θ* line indicating the value of *θ* that maximizes the QFI_*B*_ for each Ω, and the blue solid line represents the optimal Ω line indicating the value of Ω that maximizes the QFI_*B*_ for each *θ. B* = 1 and Γ_0_ = 0.03*B*.

As an example, we first plot QFI_*B*_ as functions of direction *θ* and coupling Ω with *N* = 10 in Fig. 2. We can see from Fig. 2 that there exists a region (the red area) in which the QFI_*B*_ is much larger than that in any other regions. And within the red area, we can find the reddest point denoted by (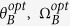), at which the QFI_*B*_ is the largest in the whole *θ* − Ω parameter space, denoted by 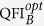. Through our numerical calculations, we find that 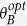 is always equal to *π*/2 but 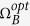 decreases with the number of spins *N*.

**Figure 3:**
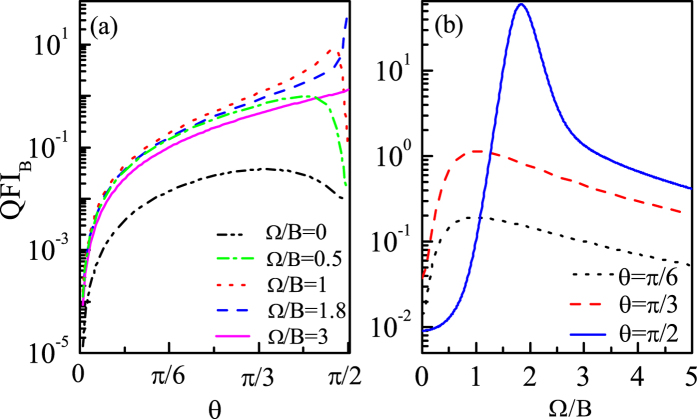
(**a**) QFI_*B*_ as a function of *θ* for different couplings (**b**) QFI_*B*_ as a function of Ω for different *θ. N* = 10, *B* = 1 and Γ_0_ = 0.03*B*.

Besides, for each fixed coupling Ω, different *θ* leads to different value of QFI_*B*_. And we plot the optimal *θ* line (black dashed line) in Fig. 2, which indicates the best direction *θ*, denoted by 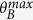, that maximizes the QFI_*B*_ for each Ω. From this optimal *θ* line we can see that at the beginning 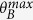 increases with Ω gradually, and when Ω increases to a certain value, 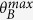 increases to *π*/2, i.e., 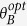, and then remains unchanged as Ω increases further. Furthermore, in order to show how the QFI_*B*_ changes along this optimal *θ* line generally, we plot the QFI_*B*_ as a function of *θ* for some different couplings with *N* = 10 in Fig. 3(a). And we can see that with Ω increasing, the maximum value of QFI_*B*_, which corresponds to 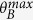, gradually increases, and when Ω increases to a certain value Ω = 1.8*B* (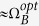), the maximum value of QFI_*B*_ almost reaches the largest, i.e., 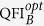, and the corresponding 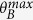 is just the optimal direction 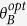 (see Fig. 2). Afterwards, as Ω increases further, although the best direction 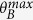 remains *π*/2, the maximum value of QFI_*B*_ decreases instead.

Meanwhile, for each fixed direction *θ*, different Ω leads to different value of QFI_*B*_. And we plot the optimal Ω line (blue solid line) in Fig. 2, which indicates the best coupling Ω, denoted by 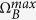, that maximizes the QFI_*B*_ for each *θ*. And from this optimal Ω line we can see that when *θ* is not close to *π*/2, 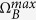 increases very slowly with *θ*, however, when *θ* is close to *π*/2, 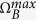 increases quickly with *θ*, finally when *θ* increases to *π*/2, 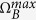 increases to 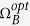. Also, in Fig. 3(b), we plot the QFI_*B*_ as a function of Ω for different directions with *N* = 10. And we can see that with *θ* increasing, the maximum value of QFI_*B*_, which corresponds to 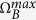, gradually increases, and when *θ* increases to *π*/2, the maximum value of QFI_*B*_ reaches the largest, i.e., 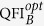, and the corresponding 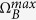 is just the optimal coupling 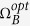 (see Fig. 2).

Now we would give physical interpretations of the above phenomena qualitatively. Firstly, that the optimal direction is 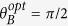 can be understood from the fact that: when *θ* = 0, there is no driving (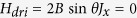), so the probe would eventually decay to the ground state |*J*, −*J*〉 (*M* = −*J*) no matter what the value of Ω is without any information of parameter *B*; when *θ* > 0, the driving strength is nonzero and increases with *θ*, so the value of QFI_*B*_ increases with *θ* either. When 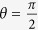, the driving strength reaches its largest value which makes the probe get populated in more energy levels, so the probe possesses more information of parameter *B*. Secondly, the estimation precision is in fact determined by a comprehensive effect among the energy level difference (*E*_*M*_ − *E*_*M*−1_), the driving strength (2*B* sin *θ*) and the decoherence rate (Γ_0_). As a result, when *θ* (Ω) is fixed, we can tune the other quantity Ω (*θ*) to its best value and hence to balance the energy level difference, the driving strength and the decoherence rate to the best, making the QFI_*B*_ the largest for the given *θ* (Ω), which is the origin of above optimal *θ* (Ω) line.

**Figure 4:**
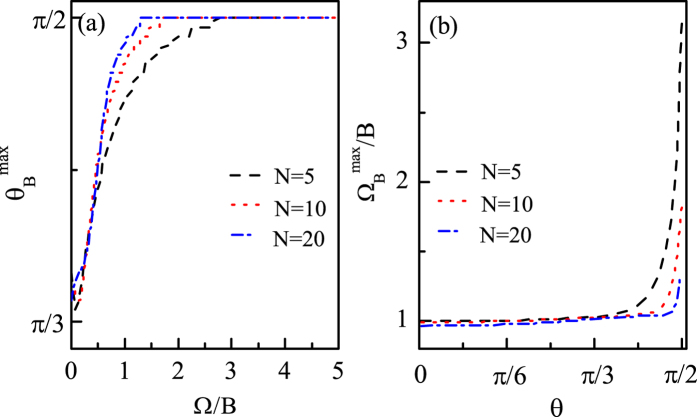
(**a**) 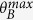 as a function of Ω for different *N* (**b**) 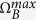 as a function of *θ* for different *N. B* = 1 and Γ_0_ = 0.03*B*.

And in Fig. 4 we show how the optimal *θ* (Ω) line changes with *N*. We can see from Fig. 4(a) that the overall profiles of the optimal *θ* line are very similar, that is, the best direction 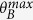 increases with Ω gradually and finally reaches 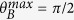 and remains unchanged, and the only difference is that with Ω increasing, the larger the *N*, the sooner the 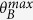 first reaches *π*/2, i.e., 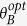. In other words, the larger the *N*, the smaller the optimal coupling 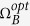, corresponding to 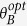. And for 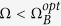, 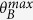 increases with *N* generally. From Fig. 4(b) we can see that the overall profiles of the optimal Ω line for different *N* are also very similar, that is, the best coupling 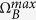 increases with *θ* very slowly when *θ* is not close to *π*/2 and increases quickly when *θ* is close to *π*/2, and finally reaches the optimal coupling, i.e., 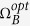, when *θ* increases to *π*/2. However, for each fixed *θ*, 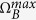 decreases with *N*, especially when *θ* is larger. And when *θ* = *π*/2 we can see that the corresponding optimal coupling 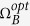 decreases with *N* obviously.

In what follows, we are interested in what the precision scaling can our probe arrive at under the correlated dissipative noise. And we find that the scaling is generally dependent on the coupling Ω, and for each direction *θ*, with a proper coupling, the scaling can be better than the HL. Here we emphasize that the scaling would eventually become worse when *N* is larger than the threshold value *N*_*c*_, which is because that when *N* is very large, the collective decoherence rate becomes very large, such that the effect of the driving can no longer compete with the decoherence. Through our numerical calculations we find that Γ_0_*N*_*c*_ ~ constant, i.e., the smaller the Γ_0_, the larger the *N*_*c*_, however, the scaling is independent of the value of Γ_0_.

**Figure 5:**
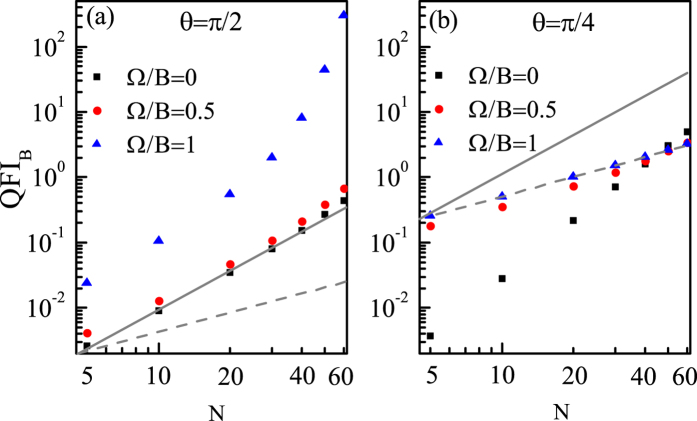
QFI_*B*_ as a function of *N* for different couplings for (**a**) *θ* = *π*/2 and (**b**) *θ* = *π*/4. The SQL (lower gray dashed line) and HL (upper gray solid line) are shifted a certain amount for a clear reference. *B* = 1 and Γ_0_ = 0.03*B*.

**Figure 6:**
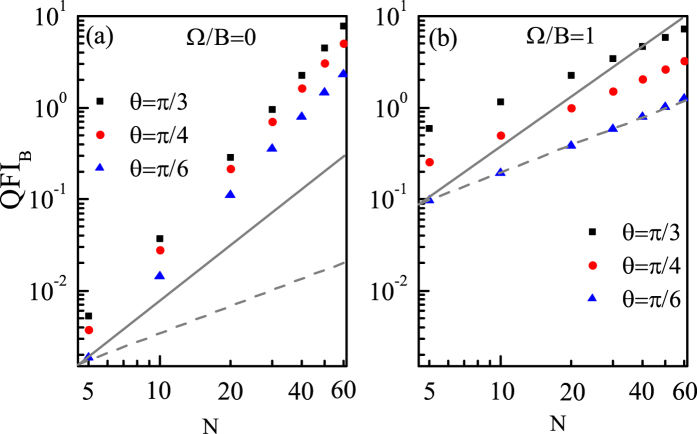
QFI_*B*_ as a function of *N* for different *θ* for (**a**) Ω/*B* = 0 and (**b**) Ω/*B* = 1. The SQL (lower gray dashed line) and HL (upper gray solid line) are shifted a certain amount for a clear reference. *B* = 1 and Γ_0_ = 0.03*B*.

As an example, we first plot QFI_*B*_ as a function of *N* for different coupling Ω with *θ* = *π*/2 and *θ* = *π*/4 in Fig. 5(a,b), respectively. We can see from Fig. 5(a) that when *θ* = *π*/2, the scaling is dependent on the value of Ω and when Ω/*B* = 0, Log(QFI_*B*_) is a linear function of Log(*N*) and the scaling is approximately equal to the HL. However, with Ω increasing, Log(QFI_*B*_) becomes gradually nonlinear with Log(*N*), accordingly the scaling becomes even better than the HL. The physical intuition behind this phenomenon is as follows: In the balance between the driving and dissipation, the lower energy levels *E*_*L*_ (*M* ~ −*J*) play a leading role. And from Eq. (4) we can obtain the energy level difference 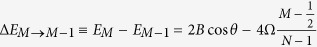, and from this expression we find that the difference between any two adjacent lower energy levels *E*_*L*_, i.e., 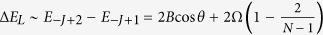, decreases with *θ*. As a result, when *θ* = *π*/2, Δ*E*_*L*_ is the smallest and the driving strength 2*B* sin *θ* is the largest, which makes the probe easier to be driven to higher energy levels. And we can see from Fig. 5(a) that the non-polynomial scaling is especially obvious at Ω/*B* = 1, this is because that when Ω/*B* = 1, the driving strength 2*B* is close to resonance with 
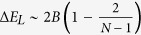
 especially for large *N*, which makes the probe the most sensitive to the parameter and the scaling become non-polynomial. While for other directions, we can see from Fig. 5(b) that the scaling is also dependent on the value of Ω, but Log(QFI_*B*_) is always a linear function of Log(*N*) no matter what the value of Ω is. Specifically, when Ω/*B* = 0, the scaling is better than the HL, but with Ω increasing, the scaling gradually becomes worse and decays approximately to the SQL when Ω/*B* = 1. And in Fig. 6 we further illustrate that when *θ* is not equal or not close to *π*/2, the scaling is the same for different direction and fixed Ω. In Fig. 6(a) we can see that when Ω/*B* = 0, the scalings for different *θ* are the same and are better than the HL. Similarly, in Fig. 6(b) we can see that when Ω/*B* = 1, the scalings for different *θ* are still the same and are approximately equal to the SQL. The reason why the scaling for Ω/*B* = 0 is better than that for Ω/*B* = 1 can be understood as follows: When *θ* is away from *π*/2, 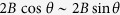, where 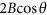 represents the modification of lower energy level difference caused by the magnetic field (i.e., the first term in 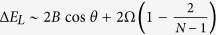), and 2*B* sin *θ* denotes the driving strength. Based on this, we can obtain that the larger the Ω, the larger the Δ*E*_*L*_, and hence the larger the detuning between Δ*E*_*L*_ and 2*B* sin *θ*, especially for large *N*, which makes the probe less sensitive to the parameter and the scaling become worse.

### Estimating direction *θ*

We now proceed to estimate the direction of magnetic field *θ*. We begin with studying which direction can be detected more accurately and what is the effect of coupling Ω on the QFI of estimating *θ*, i.e., QFI_*θ*_. According to Eq. (12) (see Methods), from our numerical calculations, we find that all the directions 0 ≤ *θ* ≤ *π*/2 can be detected. In particular, our probe is significantly sensitive to the direction *θ* ~ *π*/2 compared with other directions.

**Figure 7:**
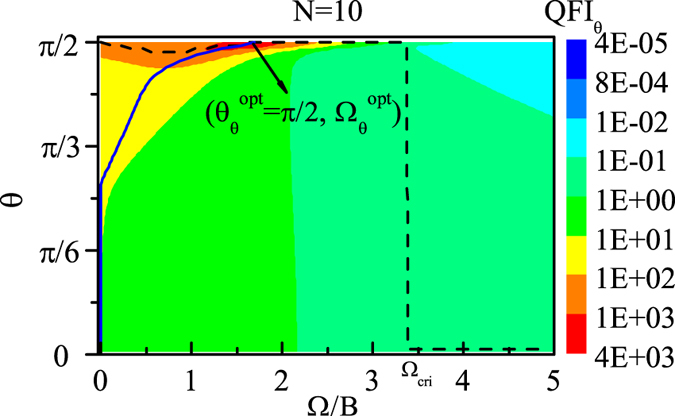
QFI_*θ*_ as functions of the direction *θ* and coupling Ω with *N* = 10. The black dashed line represents the optimal *θ* line indicating the value of *θ* that maximizes the QFI_*θ*_ for each Ω, and the blue solid line represents the optimal Ω line indicating the value of Ω that maximizes the QFI_*θ*_ for each *θ. B* = 1 and Γ_0_ = 0.03*B*.

As an example, we first plot QFI_*θ*_ as functions of direction *θ* and coupling Ω with *N* = 10 in Fig. 7. We can see from Fig. 7 that all the directions can be detected and are indeed affected by the coupling Ω more or less. In particular, we can see that there exists a region (the red area) whose values of QFI_*θ*_ are much larger than that of any other regions. Moreover, we can see that this red area corresponds to the direction *θ* ~ *π*/2, which means that our probe can detect the direction *θ* ~ *π*/2 with a very high precision. Especially when Ω is set as the optimal coupling 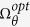, the direction *θ* = *π*/2 can be detected with the highest precision (the reddest point), denoted by 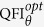, and this optimal direction is written as 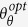.

**Figure 8:**
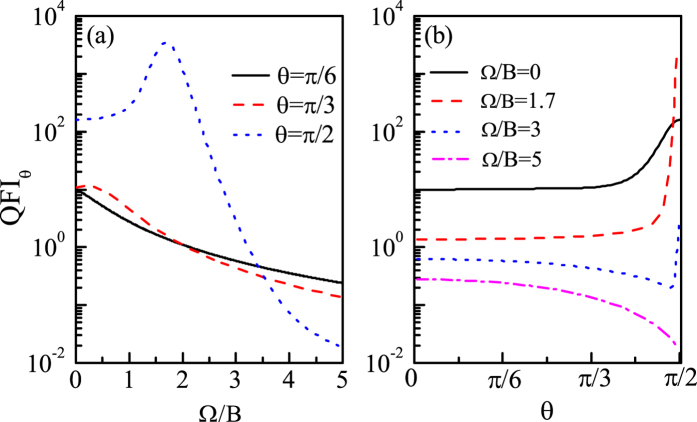
(**a**) QFI_*θ*_ as a function of Ω for different directions (**b**) QFI_*θ*_ as a function of *θ* for different Ω. *N* = 10, *B* = 1 and Γ_0_ = 0.03*B*.

Of course for any other direction *θ*, there also exists a corresponding best coupling 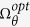, which maximizes the QFI_*θ*_ for each direction *θ*. This is manifested with a blue solid line in Fig. 7, called the optimal Ω line. And we can see from this optimal Ω line that with *θ* increasing, 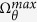 is always equal to zero at the beginning and then gradually increases with *θ*, and when *θ* increases to *π*/2, 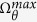 increases to 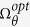. In order to show how the QFI_*θ*_ changes along this optimal Ω line, we plot the QFI_*θ*_ as a function of Ω for different directions with *N* = 10 in Fig. 8(a). And we can see that with *θ* increasing, the maximum value of QFI_*θ*_, which corresponds to 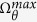, gradually increases, and when *θ* increases to *π*/2, the maximum value of QFI_*θ*_ reaches the largest, i.e., 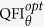, and the corresponding 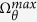 is just the optimal coupling 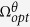.

Besides, we also investigate which direction can be detected the most accurately for each given coupling, denoted by 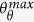, and plot the optimal *θ* line (black dashed line) in Fig. 7. The optimal *θ* line shows the best direction 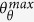 for each coupling Ω. And we can see from the optimal *θ* line that there exists a critical coupling Ω_*cri*_, and when Ω < Ω_*cri*_, 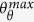 is equal or close to *π*/2; when Ω < Ω_*cri*_, 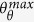 suddenly changes to 0. This can be understood as follows: Firstly, we should emphasize that the value of QFI_*θ*_ is strongly dependent on the driving strength and the sensitivity to the direction *θ*. Specifically, when Ω is large, the second term in 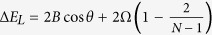 becomes large, so that the first term 2*B* cos *θ* becomes relatively small with respect to the the second one. As a result, the modification of 2*B* cos *θ* on Δ*E*_*L*_ can be ignored. However, the driving ‘*H*_*dri*_ = 2*B* sin *θJ*_*x*_’ is the most sensitive to the direction *θ* = 0 (the gradient of 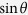 is the largest at *θ* = 0), so the best direction is 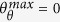 for large Ω. In contrast, when Ω is small, the second term in Δ*E*_*L*_ also becomes small so that the first term becomes relatively larger compared with the second one and hence the modification of the first term 2*B* cos *θ* on Δ*E*_*L*_ becomes prominent. Additionally, due to the fact that 2*B* cos *θ* is the most sensitive to the direction *θ* = *π*/2 (the gradient of 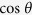 is the largest at *θ* = *π*/2), which is also the direction that makes the driving strength the largest, the best direction for relatively small Ω is 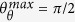.

In Fig. 8(b), we plot the QFI_*θ*_ as a function of direction *θ* for (a) Ω = 0 (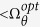), (b) Ω = 1.7*B* (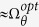), (c) Ω = 3*B* (
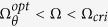
) and (d) Ω = 5*B* (>Ω_*cri*_) with *N* = 10. We can see from Fig. 8(b) that when Ω < Ω_*cri*_, the best directions are all equal to *π*/2 and the corresponding QFI_*θ*_ for *θ* = *π*/2 are much larger than that for other directions, and the closer the coupling Ω is to 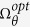, the more accurately we can detect the direction *θ* = *π*/2; when 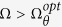 and Ω is close to Ω_*cri*_ (Ω_*cri*_ ≈ 3.384*B* for *N* = 10) the difference of QFI_*θ*_ between *θ* = *π*/2 and *θ* = 0 becomes smaller; when Ω > Ω_*cri*_, the best direction has already changed to *θ* = 0 and the corresponding value of QFI_*θ*_ is much smaller than that of the case Ω < Ω_*cri*_, because in this case although the driving is very sensitive to the direction *θ* ~ 0 as explained above, the driving strength is very small, which is bad for a large value of QFI_*θ*_.

**Figure 9:**
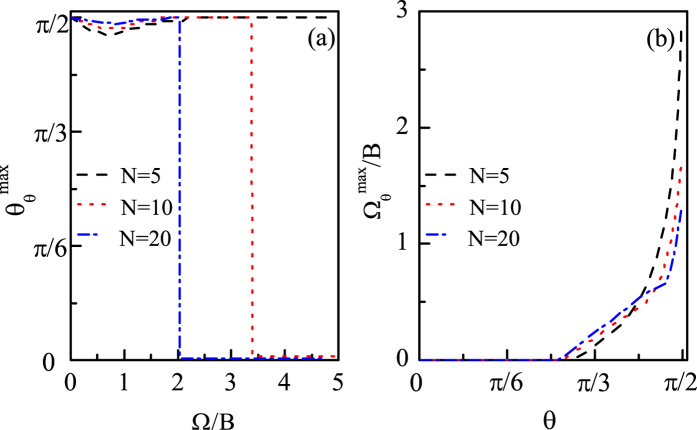
(**a**) 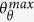 as a function of Ω for different *N* (**b**) 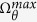 as a function of *θ* for different *N. B* = 1 and Γ_0_ = 0.03*B*.

And in Fig. 9 we show how the values of Ω_*cri*_ and Ω_*opt*_ change with *N*. We can see from Fig. 9(a) that Ω_*cri*_ decreases with *N*, and when Ω < Ω_*cri*_ all the best directions 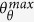 are much closer to *π*/2 for larger *N*. From Fig. 9(b) we can see that with *θ* increasing, 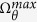 for different *N* are all equal to 0 at the beginning and then become different gradually. And when *θ* increases to *π*/2, the corresponding optimal coupling 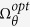 decreases with *N* obviously.

**Figure 10:**
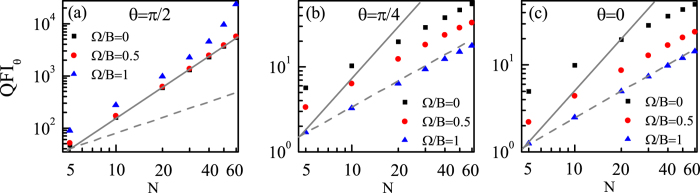
QFI_*θ*_ as a function of *N* for different couplings for (**a**) *θ* = *π*/2, (**b**) *θ* = *π*/4 and (**c**) *θ* = 0. The SQL (lower gray dashed line) and HL (upper gray solid line) are shifted a certain amount for a clear reference. *B* = 1 and Γ_0_ = 0.03*B*.

In what follows, we would investigate the precision scaling for estimating *θ* under the correlated noise. Through our numerical calculations, we find that when *θ* is equal or close to *π*/2, the precision scaling can be approximately equal to the HL for Ω/*B* = 0 or even better than the HL for Ω/*B* > 0. While for other directions, the precision scaling is constrained near the SQL. As an example, we plot the QFI_*θ*_ as a function of *N* for different coupling Ω with *θ* = *π*/2, *θ* = *π*/4 and *θ* = 0 respectively in Fig. 10. We can see from Fig. 10(a) that when *θ* = *π*/2, the scaling is dependent on the value of Ω, and when Ω/*B* = 0, Log(QFI_*θ*_) is a linear function of Log(*N*) and the scaling is approximately equal to the HL. However, with Ω increasing, Log(QFI_*θ*_) becomes gradually nonlinear with Log(*N*) and the scaling becomes better than the HL. While for other directions, we can see from Fig. 10(b,c) that Log(QFI_*θ*_) is always a linear function of Log(*N*) and the scaling is the same for different couplings, only constrained near the SQL. And the reason why the scaling for *θ* = *π*/2 is better than that for other directions can be understood as follows: Firstly, we emphasize that with *N* increasing, the number of energy levels increases, and thus the parameter information can be distributed in more energy levels, which is advantageous for the estimation. But due to the existence of dissipation, the lower energy levels would play a more important role than the higher energy levels in the estimation. Specifically, when *θ* = *π*/2, the lower energy level difference 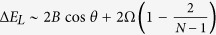 is smaller and the driving strength 2*B* sin *θ* is larger, therefore the probe is easier to be driven to more higher energy levels, and possesses more information of *θ*. In contrast, when *θ* is much smaller than *π*/2, Δ*E*_*L*_ becomes larger, and the driving strength becomes smaller, and thus the probe would be harder to be driven to higher energy levels despite of the increased energy levels resulted from the increasing *N*. As a result, the scaling for *θ* = *π*/2 is better than that for *θ* < *π*/2.

## Discussion

In summary, we have investigated the magnetic field sensing in the presence of a correlated dissipative Markovian noise. We have used a ring spin chain consisting of *N* spins, with only the nearest neighbor interactions, as our probe to estimate both the intensity *B* and the direction *θ* of magnetic field when the probe reaches its steady state.

On the one hand, for estimating *B*, we have found that the coupling between spins in the probe plays an important role in the precision, and the largest value of QFI can be achieved when *θ* = *π*/2 together with an optimal coupling, whose value decreases with the number of spins *N*. This is because that when *θ* = *π*/2, the driving caused by the external magnetic field reaches its largest value which makes the probe get populated in more energy levels, so the probe possesses more information of parameter *B*. In addition, the estimation precision is in fact determined by a comprehensive effect among the energy level difference, the driving strength and the decoherence rate. As a result, when *θ* = *π*/2, i.e., the driving strength is fixed to its largest value, we can tune the coupling to an optimal value to balance the energy level difference, the driving strength and the decoherence rate to the best, to make the QFI the largest. We have also investigated the precision scaling for estimating *B*, and found that the scaling is dependent on the coupling and for any given direction *θ*, the precision scaling with a proper coupling can be better than the HL.

On the other hand, for estimating *θ*, we have found that the coupling also plays an important role in the precision, moreover, the value of QFI is strongly dependent on *θ*. Specifically, our probe can perform a very high precision detection for *θ* ~ *π*/2, with the QFI much larger than that for any other directions, especially when the coupling is tuned to the optimal value. That is, the probe can be much more sensitive to the direction *θ* ~ *π*/2 than any other directions. And we have also investigated the precision scaling for estimating *θ*, and found that the scaling is also dependent on *θ*. When *θ* ~ *π*/2, the precision scaling can be better than the HL, but for other directions, the precision scaling is only limited to the SQL no matter what the value of coupling is.

Our results on precision scaling for estimating both *B* and *θ* are in stark contrast with the previous works on correlated dephasing noise, whether for the dynamical estimation where the best scaling is limited below the SQL^38^ or for the steady state estimation where the scaling is only the SQL^42^. Here, we emphasize that the better than SQL scalings presented in this paper are determined by a comprehensive effect of the correlated noise, the driving and the energy level difference. Specifically, if there is no driving, the correlated noise would cascade the probe to the ground state, leaving the steady state without any information of the parameter. In this case the noise is detrimental to estimation. However, if the driving is applied, which is something similar to optical pumping, the probe would populated in not only the ground state, and now the correlated noise as a common bus, could create some correlations in the steady state, making the QFI or its scaling better. But whether it is due to the entanglement is still ambiguous for us. In fact, we have calculated the entanglement quantified by concurrence for the stationary state with *N* = 2 and compared the value of QFI with the concurrence. And we have found that the correlation between the QFI and the concurrence is very complicated. For instance, when Ω/*B* = 0, the best direction with the largest QFI has only zero concurrence and for some other directions the concurrence does not equal 0, which means that the largest concurrence does not correspond to the largest QFI. However, when Ω/*B* > 0, for estimating intensity *B*, the larger the concurrence, the larger the QFI except for *θ* = *π*/2, while for estimating *θ*, the largest QFI does not correspond to the largest concurrence. Actually it has been shown in ref. 50 that as the entanglement of a state decreases, its QFI can even increase. So it is hard to say whether the high precision or the better-than-SQL scalings are due to the entanglement in the stationary state. However, correlations existing in the stationary state do work for the better scalings. This can be understood from the following view: When Ω/*B* = 0, i.e., there are no interactions in the Hamiltonian, the scaling is only the SQL for the independent noise, because the steady state is a product state. As we know that in general, there are two types of two qubit couplings: direct coupling, for example, dipole coupling in NMR or Coulomb coupling in superconducting charge qubits, and indirect coupling mediated by another quantum system like a harmonic oscillator or even a common environment called common bus. For our correlated noise, due the indirect interactions mediated by this common bus, some correlations between the qubits in the probe might be produced, i.e., the steady state is not a product state any more, and the scaling for Ω/*B* = 0 can even be better than the SQL, which implies that some correlations created by the correlated noise in the steady state play an indispensable role in the better scalings.

On the other hand, it should be noted that the better scalings presented in this paper are not related to quantum phase transitions, because from our numerical calculations we have found that the QFI of the steady state at the critical point for our model has no singularity. Actually, the study of the connection between the quantum phase transitions and the behavior of QFI is focused on the ground state, and it has been shown that at the critical point, the QFI would make a significant enhancement. This is because that on the two sides of critical point of quantum phase transitions, the property of the ground states changes qualitatively, and the QFI describes how well small change of a parameter can be probed. Therefore, the QFI of the ground state with respect to the external parameter inducing the quantum phase transitions can be highly enhanced. However, in our case, we have used the steady state to estimate a certain parameter, not the ground state, so all the energy levels which are populated by the probe would contribute to the estimation precision.

Besides, the results presented in this paper are based on the QFI which can be achieved when the optimal measurement, consisting of a set of projectors on the eigenbasis of the the symmetric logarithmic derivative *L*_*ρ*_, is applied. However, generally *L*_*ρ*_ does not correspond to an observable quantity, so how to find a realistic feasible measurement scheme which can make the precision equal to the QFI deserves our investigation. Through our numerical calculations, we have found that the *J*_*z*_ basis is approximately the optimal measurement when *θ* = 0 or *π*/2. That is, the classical Fisher information for *J*_*z*_ measurement basis is approximately equal to the QFI when *θ* = 0 or *π*/2. While for other directions, the optimal measurement is approximately the total angular momentum *J*_**n**_ basis (**n** represents the specific direction of the total angular momentum), which depends on the direction of the magnetic field.

## Methods

Our results are based on the parameter estimation theory. And the task of parameter estimation is to determine a parameter from a set of output data. In quantum metrology, one estimates a parameter *x* by measuring a parameter dependent state, say *ρ*_*x*_, which is evolved from a suitable initial state *ρ*(0), afterwards, one would choose an unbiased estimator 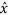 to process the measurement output data and infer the unknown parameter *x*. An appropriate measure to quantify the performance of estimation strategy is the standard deviation of this estimator, 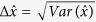, which is lower bounded by the Cramér-Rao bound as follows:


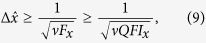


where *ν* is the number of independent experimental repetitions, *F*_*x*_ is the Fisher information, which optimizes over all the possible estimators, and QFI_*x*_ is the quantum Fisher information, which optimizes over all the allowable measurements and is given by


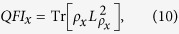


where the symmetric logarithmic derivative 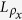 in the above equation is defined as:


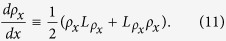


Writing *ρ*_*x*_ in its spectral decomposition as *ρ*_*x*_ = ∑_*i*_*p*_*i*_|*ψ*_*i*_〉〈*ψ*_*i*_|, one can obtain:


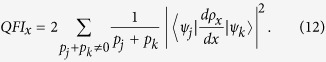


## Additional Information

**How to cite this article**: Guo, L.-S. *et al*. Magnetic field sensing subject to correlated noise with a ring spin chain. *Sci. Rep.* **6**, 33254; doi: 10.1038/srep33254 (2016).
